# Transcriptomic Profiling of DAF-7/TGFβ Pathway Mutants in *C. elegans*

**DOI:** 10.3390/genes11030288

**Published:** 2020-03-09

**Authors:** Muhan Hu, David Crossman, Jeevan K. Prasain, Michael A. Miller, Rosa A. Serra

**Affiliations:** 1 Department of Cell Development and Integrative Biology, University of Alabama at Birmingham, Birmingham, AL 35294, USA; mamiller@uab.edu (M.A.M.); rserra@uab.edu (R.A.S.); 2 Department of Genetics, University of Alabama at Birmingham, Birmingham, AL 35294, USA; dkcrossm@uab.edu; 3 Department of Pharmacology and Toxicology, University of Alabama at Birmingham, Birmingham, AL 35294, USA; jprasain@uab.edu

**Keywords:** TGFβ, *daf-1*, *daf-3*, RNA sequencing, *C. elegans*, gene ontology, qPCR

## Abstract

The transforming growth factor beta superfamily encompasses a large family of ligands that are well conserved across many organisms. They are regulators of a number of physiological and pathological processes. The model nematode *Caenorhabditis elegans* has been instrumental in identifying key components of the transforming growth factor beta (TGFβ) pathway. In *C. elegans*, the TGFβ homolog DAF-7 signals through the DAF-1 Type I and DAF-4 Type II receptors to phosphorylate downstream R-SMADs DAF-8 and DAF-14. These R-SMADs translocate into the nucleus to inhibit Co-SMAD DAF-3. Many of the roles of the canonical DAF-7 pathway, involving both DAF-1 and DAF-3, have been identified using targeted genetic studies. Few have assessed the global transcriptomic changes in response to these genes, especially in adult animals. In this study, we performed RNA sequencing on wild type, *daf-1*, and *daf-1*; *daf-3* adult hermaphrodites. To assess the overall trends of the data, we identified differentially expressed genes (DEGs) and performed gene ontology analysis to identify the types of downstream genes that are differentially expressed. Hierarchical clustering showed that the *daf-1*; *daf-3* double mutants are transcriptionally more similar to wild type than *daf-1* mutants. Analysis of the DEGs showed a disproportionally high number of genes whose expression is increased in *daf-1* mutants, suggesting that DAF-1 acts as a general repressor of gene expression in wild type animals. Gene ontology analysis of the DEGs produced many significantly enriched terms, including Molting Cycle, Response to Topologically Incorrect Protein, and Response to Biotic Stimulus. Understanding the direct and indirect targets of the DAF-7 TGFβ pathway through this RNA-seq dataset can provide insight into novel roles of the multifunctional signaling pathway, as well as identify novel genes that may participate in previously reported functions of TGFβ signaling.

## 1. Introduction

Transforming growth factor beta (TGFβ) is the prototype of the large family of secreted ligands that act through downstream receptors to modulate diverse biological functions, such as embryological development, adult stem cell differentiation, immune regulation, wound healing, inflammation, and cancer [1,2,3,4,5,6]. The functionality of this family of proteins is highly conserved among many organisms, including human, mice, nematodes, and flies [7,8,9]. Canonically, TGFβ signals as homo- and heterodimers to activate serine/threonine kinase receptors that act as heterotetramers to phosphorylate downstream receptor-regulated SMADs (R-SMADs). The activated R-SMADs then translocate into the nucleus to regulate the transcription of a wide array of downstream targets [10]. Studies in the past three decades have provided great insight into the multifunctionality of the TGFβ pathway in affecting developmental and pathologic mechanisms, revealing different, and sometimes opposite, responses to TGFβ. For example, while TGFβ has been shown to inhibit the growth of many cell types, such as epithelial [11,12], hematopoietic [13,14], and glial cells [15,16], it can also stimulate the growth of other cell types, such as chondrocytes and osteoblasts [17,18]. These studies unveil the complexities of this pathway and suggest that identification of key effectors of the pathway is crucial to teasing out the mechanisms underlying the differential effects of TGFβ.

**Figure 1:**
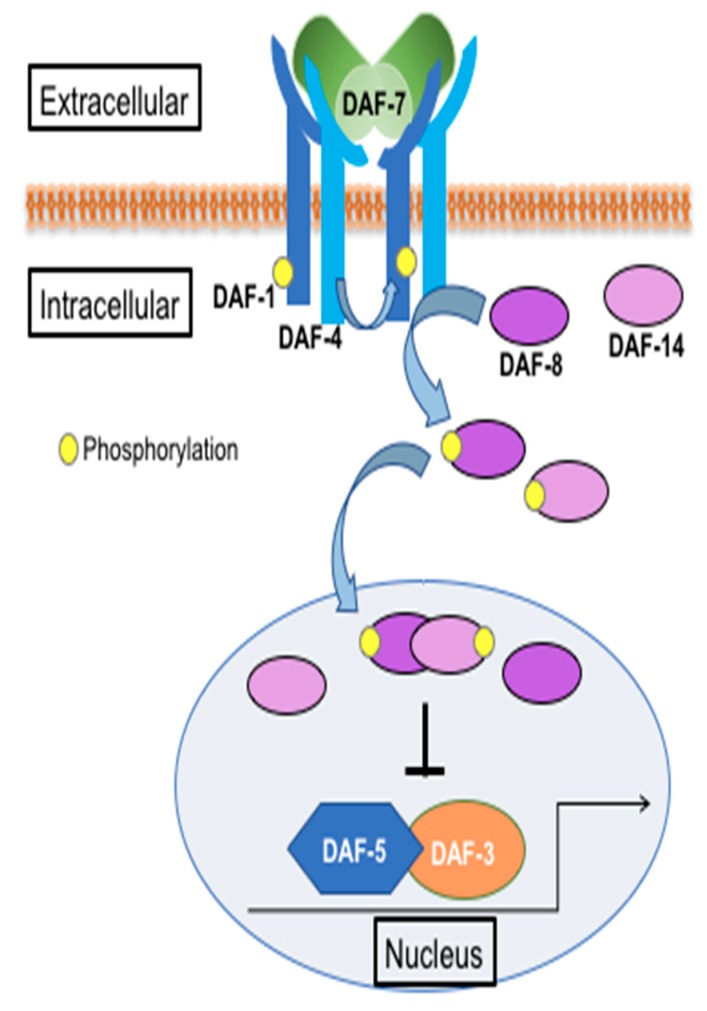
Schematic of the DAF-7/TGFβ pathway in *C. elegans.* (Adapted from wormbook.org).

Genetic studies in *Caenorhabditis elegans* have been instrumental in the identification of key TGFβ pathway components. In *C. elegans*, DAF-7 encodes a TGFβ homolog and is known primarily for its role in regulating dauer formation [19]. DAF-7 is secreted from the ASI sensory neurons in response to environmental cues, such as food, pheromones, and pathogens [20,21]. It acts as a homodimer to bind the DAF-1 Type I and DAF-4 Type II ser/thr kinase receptors. These receptors function as heterotetramers to phosphorylate downstream R-SMADs DAF-8 and DAF-14. The activated SMADs translocate to the nucleus to suppress the Co-SMAD DAF-3 (Figure 1) [8]. The *daf* genes were initially identified for their roles in dauer formation. They are divided into two categories: dauer constitutive (e.g., *daf-7*, *daf-1*, *daf-4*, *daf-8*, and *daf-14*) and dauer defective (*daf-3*). Genetic crosses of dauer constitutive and dauer defective mutants result in double mutants, such as *daf-1*; *daf-3*, that are dauer defective, suggesting that *daf-3* is epistatic to *daf-1*. This relationship has been observed in many other functions of the pathway, some of which may be conserved in higher animals, including humans.

In addition to dauer formation, DAF-7 in *C. elegans* has been shown to play a role in regulating the expression of AMPA-type glutamate receptor GLR-1 [22], the expression of chemoreceptors in amphid neurons [23], the expression of genes in the distal tip cell to modulate the germline stem cell niche [24,25], and the production of prostaglandins that are important for sperm guidance [26]. These studies illustrate that, similar to mammalian systems, *C. elegans* TGFβ regulates a wide array of biological functions. Understanding the direct and indirect targets of this pathway may uncover novel functions and regulatory mechanisms that are translatable to mammalian systems.

Many of the known targets downstream of DAF-1 and DAF-3 have been identified via direct genetic studies or forward genetic screens. Few studies have characterized the transcriptional profiles of adult TGFβ mutants [27]. In this study, we performed RNA sequencing on wild-type, *daf-1*, and *daf-1*; *daf-3* mutants. We looked at the general trends of the RNA sequencing data, focusing on the genes that are differentially altered by *daf-1* and *daf-3*. The datasets provided in this study are some of the first to be generated by RNA sequencing of the DAF-7 TGFβ pathway mutants. They provide a tool that can facilitate future work in identifying DAF-3 dependent and independent functions and downstream factors of the pathway in *C. elegans*.

## 2. Materials and Methods

### 2.1. C. elegans Strains and Maintenance

*C. elegans* strains Bristol N2 (wild type), *daf-1*(*m40*), and *daf-3*(*mgDf90*) were ordered from the Caenorhabditis Genetics Center (CGC), which is funded by the National Institutes of Health (NIH) Office of Research Infrastructure Programs. The *daf-1*(*m40*)*; daf-3*(*mgDf90*) double mutant strain was generated through genetic crosses. *C. elegans* strains were maintained in NA22 *Escherichia coli* bacteria on nematode growth media (NGM) plates at 16 °C or 25 °C.

To grow worms for RNA isolation, worms were first synchronized by the bleaching technique [28]. Briefly, gravid worms were recovered from ten 10 cm plates by washing with M9 buffer. Worm pellets were washed with M9 buffer until the buffer was clear of bacteria. The resulting worm pellet was exposed to an alkaline hypochlorite solution (1 mg NaOH, 25 mL sodium hypochlorite, and 74 mL H_2_O) and vortexed for 2 min. The mixture was centrifuged up to 1000× *g* for 5 s. The resulting pellet contained a white layer (eggs) and a yellow layer (intact gravid worms). The supernatant was discarded, and the process was repeated with fresh alkaline hypochlorite solution until the pellet was mostly white. The reaction was terminated by washing 5–6 times with M9 buffer. The resulting white pellet was resuspended in 6 mL of S. media and agitated overnight at 16 °C on a nutator to allow the eggs to hatch into L1 larva. The synchronized L1 larva were plated on 15 cm NGM plates and supplemented with thick NA22 *E. coli*. The synchronized worms were grown at 16 °C until the L4 stage and shifted to 25 °C for 24 h before harvesting with M9 buffer. The worms were washed with M9 buffer until the supernatant was clear of bacteria, and the worm pellets were stored in 250 mg aliquots at −80 °C.

### 2.2. RNA Isolation

250 mg of frozen, synchronized worms were homogenized with a Bullet Blender 5 (Next Advance, Troy, NY, USA) and 0.5 mm zirconium beads. Total RNA was extracted with TRIzol (Invitrogen, Carlsbad, CA, USA) following the kit’s protocol.

### 2.3. Next-Generation Sequencing on Illumina Platforms

Sequencing of the total RNA was performed at the University of Alabama at Birmingham (UAB) Heflin Center for Genomic Science Core Laboratories. mRNA-sequencing was performed on the Illumina HiSeq2500 using the latest versions of the sequencing reagents and flow cells providing up to 300 Gb of sequence information per flow cell. Briefly, the quality of the total RNA was assessed using the Agilent 2100 Bioanalyzer followed by 2 rounds of poly A+ selection and conversion to cDNA. We used the TruSeq library generation kits as per the manufacturer’s instructions (Illumina, San Diego, CA, USA). Library construction consists of random fragmentation of the polyA mRNA, followed by cDNA production using random primers. The ends of the cDNA were repaired and A-tailed, and adaptors ligated for indexing (up to 12 different barcodes per lane) during the sequencing runs. The cDNA libraries were quantitated using qPCR in a Roche LightCycler 480 with the Kapa Biosystems kit for library quantitation (Kapa Biosystems, Woburn, MA) prior to cluster generation. Clusters were generated to yield approximately 725–825 K clusters/mm^2^. Cluster density and quality were determined during the run after the first base addition parameters were assessed. We ran paired end 2 × 50 bp sequencing runs to align the cDNA sequences to the reference genome (Table S1).

### 2.4. Data Assessment

The raw FASTQ reads were first trimmed using TrimGalore! Version 0.4.0 to remove any primer adapter contamination. TopHat version 2.0.12 was then used to align the trimmed FASTQ reads to the Ensembl reference genome WBcel235.ceWS240 Release 76 (www.ensembl.org) using the short read aligner Bowtie version 2.2.3 [29,30,31]. Cufflinks version 2.2.1 with default parameters used the aligned reads from TopHat to assemble transcripts, estimate their abundances, and test for differential expression and regulation [31,32]. Cuffmerge, which is part of Cufflinks that merged the assembled transcripts to a reference annotation. Finally, Cuffdiff found significant changes in transcript expression. When looking for genes with statistically significant changes in absolute expression, Cuffdiff uses a *t*-test to calculate the *p* values of the observed changes and Benjamini-Hochberg multiple testing correction to calculate the q values.

### 2.5. Data Visualization and Gene Ontology

Principle component analysis (PCA) analysis and heatmapping of the data were performed using the fragments per kilobase of exon model per million reads mapped (FPKM) values of the individual samples in Rstudio (Version 1.1.456) [33]. PCA was calculated by inputting the normalized FPKM values into the prcompt command with Scale = TRUE and plotted using ggplot2 [34]. The heatmap was generated using heatmap.2 of z transformed FPKM values [35]. For gene ontology, differentially expressed genes (DEGs) in the *daf-1* vs. N2 dataset with ≥2 fold difference, q < 0.05 were first identified. These genes were then compared to the DEGs in the *daf-1* vs. *daf-1*; *daf-3* dataset with ≥2 fold difference, *p* < 0.05, q < 0.05. Only genes that were increased or decreased in *daf-1* when compared to both N2 and *daf-1*; *daf-3* were used for gene ontology analysis. The list of identified genes was entered into the enrichment analysis tool on wormbase.org [36]. The gene ontology terms were recorded, and parent child terms were manually identified and noted in the data tables.

### 2.6. Data Validation by qRT-PCR

The same RNA used for RNA-seq was used for quantitative real-time polymerase chain reaction (qRT-PCR) analysis. cDNAs were synthesized from 1µg of RNA using the SuperScript IV Reverse Transcriptase (Invitrogen) kit and random hexamers following the provided protocol. Real-time PCR was performed in the Bio-Rad CFX Connect Real-Time System with Syber Green master mix (Bio-Rad, Hercules, CA, USA) following the provided protocol. Each sample was run in triplicate. Three biological samples were run for N2 (wild-type), *daf-1*(*m40*)*,* and *daf-1*(*m40*); *daf-3*(*mgDf90*) double mutants. The PCR protocol used was: 95 °C for 2 min; 40 cycles of 95 °C for 5 s and 60 °C for 30 s. The primers used are listed in Table S2. The relative expression fold change was calculated in the CFX manager software system (Bio-Rad) using the 2^^−ddCT^ method. *cdc-42* was used as a control.

## 3. Results

### 3.1. Overall Trends of the Wild-Type and TGFβ Mutant Transcriptomes

**Figure 2:**
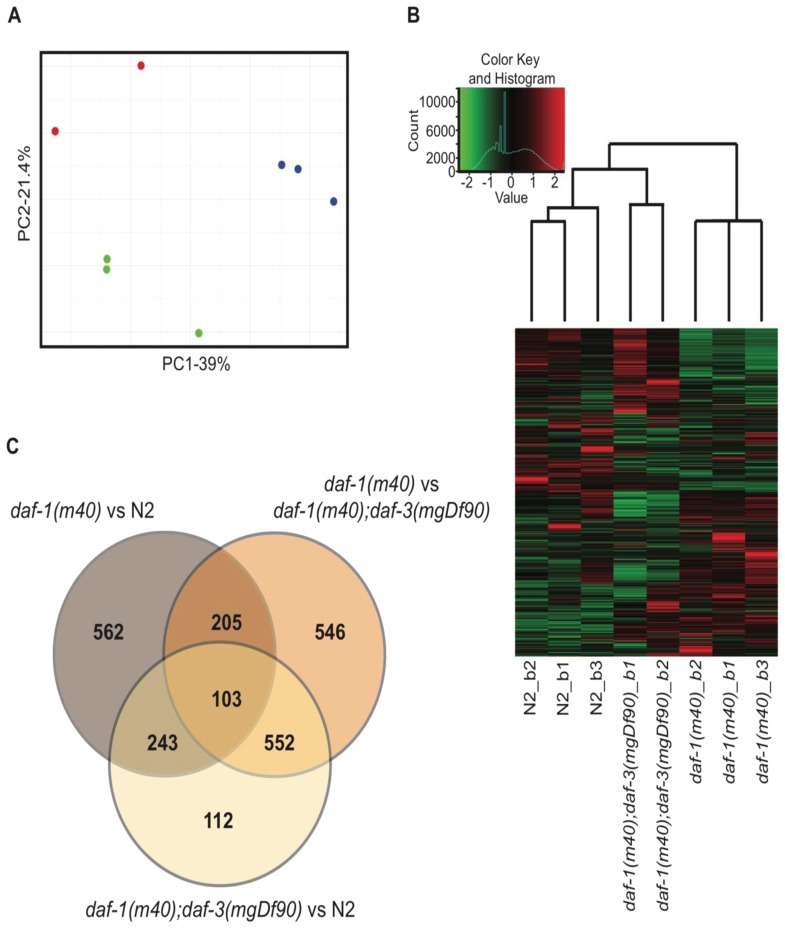
Description of the RNA-seq data. (**A**) Principle component analysis (PCA) of the sequenced samples. Red dots denote *daf-1*; *daf-3* double mutants, blue dots denote *daf-1* single mutants, and green dots denote wild-type N2. (**B**) The heatmap of the eight samples displayed in the PCA analysis (**A**). Samples are clustered hierarchically. Values on the x-axis of the color key denote relative z-scores. Count value on the y-axis denote the number of genes belonging to each z value on the x-axis. (**C**) Venn diagram of the differentially expressed genes that are ≥2-fold difference, q < 0.05 in *daf-1* vs. N2, *daf-1* vs. *daf-1*; *daf-3*, and *daf-1*; *daf-3* vs. N2.

We extracted total RNA from N2, *daf-1*, and *daf-1*; *daf-3* adult hermaphrodites. Three biological replicates (i.e., batches, denoted as b1-b3) per genotype were processed for RNA sequencing. PCA using the FPKM values of all 9 samples showed that *daf-1*; *daf-3* batch 3 was an extreme outlier (Figure S1). This sample was therefore removed from further analyses. PCA of the remaining 8 samples showed close clustering within replicates of the same genotype and a high degree of variance between genotypes (Figure 2A). Hierarchical clustering in the heatmap further confirmed that replicates of the same genotype were more similar to each other than to samples of other genotypes (Figure 2B). It was of interest that *daf-1*; *daf-3* double mutants clustered more closely with wild-type than *daf-1* mutants. This finding coincided with many functional studies showing that *daf-3* suppresses the phenotypes of *daf-1* single mutants, suggesting that that *daf-1*; *daf-3* double mutants are also functionally more similar to wild-type than *daf-1* animals.

We used Cufflinks to identify DEGs between the sequenced genotypes. Only genes that showed a ≥ 2-fold difference at q < 0.05 were identified for downstream analysis. 1113 DEGs passed these criteria in the *daf-1* vs. N2 dataset and 1406 DEGs were identified in the *daf-1* vs. *daf-1*; *daf-3* dataset (Figure 2C, Tables S3 and S4). Of these DEGs, only 308 genes overlapped between the two datasets. This suggested that while *daf-1* and *daf-3* each contributed to wide spread changes in the transcriptome of the *C. elegans*, only a small fraction of downstream targets are regulated by both *daf-1* and *daf-3*.

**Figure 3:**
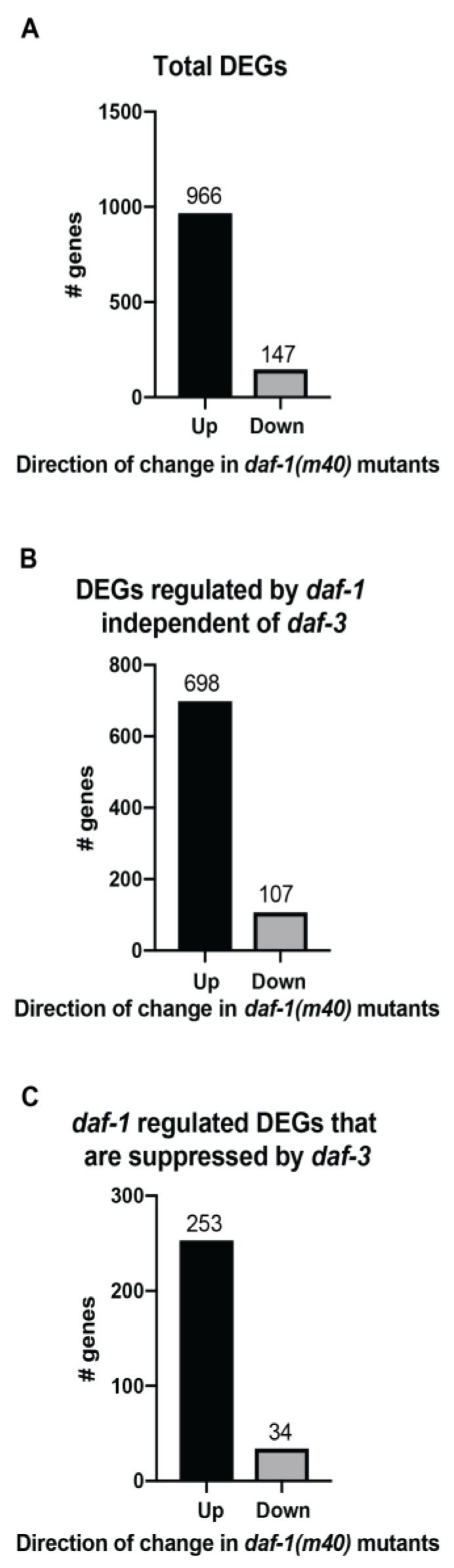
General trends of the differentially expressed genes (DEGs). The bar graphs represent the number of up and down regulated genes in *daf-1*(*m40*) mutants in (**A**) All DEGs in the *daf-1* vs. N2 dataset. (**B**) DEGs in the *daf-1* vs. N2 dataset that are regulated by *daf-1* independent of *daf-3*. (**C**) DEGs that are differentially regulated by *daf-1* and *daf-3*. The number above each bar denotes the number of genes.

To understand how *daf-1* and *daf-3* regulated their downstream targets, we assessed the number of up and down regulated genes in each dataset. Of the 1113 DEGs in the *daf-1* vs. N2 dataset, 966 were increased in *daf-1* mutants (Figure 3A), suggesting that *daf-1* acts as a general repressor of gene expression in wild-type animals. We dissected the data further into genes that were regulated by *daf-1* independent of *daf-3* (i.e., genes whose expression is altered in the *daf-1* vs. N2 dataset, but not in the *daf-1* vs. *daf-1*; *daf-3* dataset, 805 genes total) and genes that were differentially regulated by *daf-1* and *daf-3* (i.e., genes that were found to be increased in *daf-1* mutants when compared to wild-type and decreased in *daf-1*; *daf-3* double mutants when compared to *daf-1*, 287 genes total).We found that in both groups, the majority of genes were upregulated in *daf-1* mutants (Figure 3B,C). These results suggested that in the context of the *daf-1* and *daf-3* signaling pathway, *daf-3* was responsible for driving the transcription of the bulk of their downstream targets, while *daf-1* worked to decrease their expression. Functional studies of this pathway have emphasized the role of *daf-1* and *daf-3* working in sequence to regulate the bulk of the reported phenotypes. However, the transcriptomics studies presented here highlight that *daf-1* and *daf-3* share only a small percentage of downstream targets.

### 3.2. Functional Annotation of the Transcriptomic Data

Our goal in this study was to understand the function of genes downstream of the canonical DAF-7 TGFβ pathway, which involves both *daf-1* and *daf-3*. We performed GO enrichment analysis using the 287 genes that are differentially regulated by *daf-1* and *daf-3* to probe for the possible functions of these downstream genes. Table 1 contains the list of GO terms that were significantly enriched (q < 0.05). Analysis of the 34 genes that were downregulated in *daf-1* mutants showed an enrichment of the biological process Response to Biotic Stimulus. The 253 upregulated genes mapped to many more GO terms in all three major GO categories. Significantly enriched terms in the biological processes included ‘Response to Topologically Incorrect Protein Folding’, ‘Response to Biotic Stimulus’, and ‘Molting Process’. Significantly enriched parent terms under cellular components included Collagen Trimer, Extracellular Region, and Membrane. Significantly enriched terms for molecular function included Structural Constituent of Cuticle, Passive Transmembrane Transporter Activity, and Peptidase Activity. Collagen Trimer and Structural Constituent of Cuticle were two of the most highly enriched terms, at 26- and 28-fold enrichment respectively. This reflected the known role of the DAf-7 TGFβ pathway in modulating the progression of larval development, which also involves the regulation of cuticle components.

We next focused on biological process terms to tease out the type of genes annotated to each GO term, as proof of principle (Table 2). For all of the genes listed in Table 2, their functions or predicted functions were identified using WormBase. For the genes in the Molting Process, *dpy-13*, *rol-6*, *sqt-1*, and *sqt-2* are all reportedly associated with components of the cuticle and collagen. *nas-30* is a metalloproteinase and is predicted to play a role in the hatching process. The term response to Biotic Stimulus were associated with genes both up- and down- regulated in *daf-1* mutants. Of the up-regulated genes, *sodh-1*, *clec-61*, and *math-38* are associated with defense to gram positive bacterium. *irg-1* and F20G2.5 participate in defense to gram negative bacterium and the innate response. *fir-7* is a fungus induced protein, and *nlp-30* is expressed in the hypodermis and responds to both bacterial and fungal infections. Of the down-regulated genes, *ilys-5* is predicted to have lysozyme activity, *clec-218* has predicted carbohydrate binding activity and responds to gram-positive bacteria. *lys-4* has predicted lysozyme activity as well as defense to gram positive bacteria. All of the genes annotated to the response to Topologically Incorrect Protein Folding either participated or were predicted to participate in the endoplasmic reticulum unfolded protein response. *abu-1*, *abu-8*, *abu-10*, and *abu-11* are members of a family of genes called Activated in Blocked Unfolded Protein Response. *col-109* is a component of the cuticle, *pqn-74* has predicted chitin binding activity, and *clec-67* has predicted carbohydrate binding activity. *Fipr-27* is a member of the fungus-induced protein family.

### 3.3. Validation of Select RNA-Sequencing Genes by Quantitative Real-Time PCR

**Figure 4:**
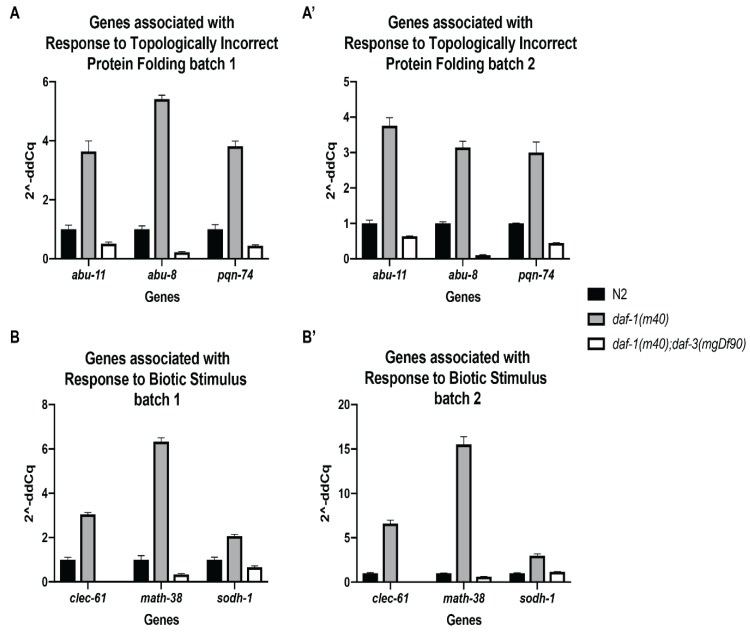
qPCR validation of select genes from the RNA-seq dataset. (**A**) Expression fold change for three Response to Topologically Incorrect Protein Folding genes. (**A’**) Biological replicate of A. (**B**) Expression fold change for three Response to Biotic Stimulus genes. (**B’**) Biological replicate of B. For all qPCR analysis, cDNA was synthesized from 1µg of total RNA. qPCR was performed using 2 µL of cDNA per reaction with gene specific primers and *cdc-42* as the endogenous control. Error bars represent standard deviation of technical triplicates.

To validate the gene expression changes observed in the RNA-seq data, we performed quantitative real-time PCR on three genes from both Response to Topologically Incorrect Protein Folding and Response to Biotic Stimulus. We used the same RNA that was submitted for RNA sequencing for the qPCR analysis. RNA from three biological replicates (i.e., batches) of N2 wild-type, *daf-1* single mutant, and *daf-1*; *daf-3* double mutant worms were assessed. *cdc-42* was selected as the only reference gene used for the validation study. We chose this gene because *cdc-42* has been a reliable reference gene used in many genetic backgrounds, and its FPKM value was approximately equal across all of our samples. Figure 4 depicts the expression fold change seen in samples from batch 1 and batch 2, corresponding to the batch numbers from the RNA-seq samples. The genes *abu-11*, *abu-8*, and *pqn-74* were selected from the Response to Topologically Incorrect Protein Folding GO term. The expression of these genes was increased in *daf-1* mutants compared to wild type, and this increase was suppressed in the *daf-1*; *daf-3* double mutants (Figure 4A,A’). A similar trend is seen with the Response to Biotic Stimulus genes *clec-61, math-38,* and *sodh-1* (Figure 4B,B’). qPCR using RNA from batch 3 of N2, *daf-1*, and *daf-1*; *daf-3* showed an increase in expression of all six genes in *daf-1*. However, a corresponding suppression of expression in the *daf-1*; *daf-3* sample was not observed for *abu-8, pqn-74*, and *sodh-1* [37], underscoring the possibility that this sample is a true outlier.

## 4. Discussion

TGFβ signaling is ubiquitously found to regulate many physiological and pathological processes and is highly conserved among many organisms [7,8,9]. In *C. elegans*, DAF-7 is a TGFβ homolog that is secreted by ASI sensory neurons in response to environmental cues, such as pheromones and food. The canonical DAF-7 TGFβ pathway signals through the DAF-1 Type I and DAF-4 Type II receptors to downstream DAF-8 and DAF-14. These R-SMADs translocate into the nucleus to inhibit the Co-SMAD DAF-3 [8,20]. This pathway has been heavily studied for its role in dauer formation and has also been implicated in other processes, such as germline development, neuronal gene expression, and fat metabolism [19,23,24,38]. While these studies have provided insight into downstream genes that are regulated by this canonical signaling mechanism, few studies have investigated the global transcriptomic profile changes that are mediated by TGFβ signaling, especially in the adult worm [27]. In this study, we performed RNA sequencing on N2, *daf-1*, and *daf-1*; *daf-3* adult hermaphrodites. We reported the general trends found in the sequencing data and focused on characterizing the transcriptomic profile of genes that are differentially regulated by both *daf-1* and *daf-3*. These genes reflect pathways in which increases or decreases in gene expression in response to *daf-1* are suppressed by *daf-3*.

Many studies on the DAF-7 TGFβ pathway reinforce the currently accepted model between *daf-3* and its upstream regulators, in which a phenotypic or genotypic change observed in *daf-1* mutants is suppressed in *daf-1*; *daf-3* double mutants, suggesting that *daf-3* is epistatic to *daf-1* [24,25,26,38]. These findings magnify the canonical DAF-7 signaling pathway and suggest that *daf-1*; *daf-3* double mutant animals are more similar to wild type animals than to *daf-1* mutants. PCA and hierarchical clustering of our RNA-seq data show that *daf-1*; *daf-3* clusters closer with N2 than *daf-1*, supporting the findings in the functional studies. However, a comparison of the DEGs between *daf-1* versus wild type and *daf-1* versus *daf-1*; *daf-3* identified only a small fraction of overlapping genes, suggesting that of the downstream genes affected by *daf-1* and *daf-3* individually, only a small fraction are co-regulated by both. This also highlights the broader functions of *daf-1* and *daf-3* outside of this canonical TGFβ signaling pathway, though we focused on co-regulated genes that are differentially regulated by *daf-1* and *daf-3* and not those regulated by *daf-1* and *daf-3* independently.

The aim of this study was to assess the profile of genes downstream of the canonical DAF-7 TGFβ pathway. Therefore, we focused the downstream GO analysis on the genes that are differentially regulated by *daf-1* and *daf-3*. The enriched GO terms reflect functions of the canonical pathway that have been shown extensively through other genetic and functional studies, as well as novel functions of the pathway that have not been well studied in *C. elegans*. For example, the canonical DAF-7 TGFβ pathway is known for its role in dauer formation. Dauer arrest is a decision made during the L1-L2 larval stages, where an animal changes its metabolism and morphology to survive harsh environmental conditions. *daf-7*, *daf-1*, *daf-14*, and *daf-8* mutants are all prone to dauer formation, a phenotype that is suppressed by *daf-3*. Most mutant alleles are temperature sensitive and must be maintained at a lower permissive temperature until the L4 larval stage to prevent dauer formation [39,40]. Dauering is a process that extensively changes the development and morphology of the animal. Correspondingly, our data showed enrichment of genes involved in the Molting Cycle, as well as the Cuticle and Collagen Components.

The canonical DAF-7 pathway has also been shown to modulate gene expression and worm behavior in response to environmental stimuli. However, few studies have looked at the mechanisms by which the canonical pathway achieves these functions. DAF-7 has been demonstrated to affect the expression of olfactory chemoreceptors on chemosensory neurons, such as the ASI, and glutamate receptor GLR-1 in the ventral nerve cord [22,23]. It has also been shown to respond to pathogenic bacteria, *Pseudomonas aeruginosa*, in addition to other cues such as food, temperature, and pheromones [19,21]. A closer inspection of the genes involved in Passive Transmembrane Transporter activity showed many predicted ion channels that are expressed in neurons and neurotransmitter transporters, including *dat-1*, *unc-8*, *unc-38*, and *twk-40*. Moreover, the GO term Response to Biotic Stimulus contained mainly genes that help the organism respond to both gram-negative and gram-positive bacteria, as well as fungi (see Table 2). These GO terms, along with their associated genes, suggest that the canonical DAF-7 TGFβ pathway may play a significant regulatory role in affecting *C. elegans* chemosensation as well as response to pathogenic versus beneficial organisms in their surroundings.

Some of the GO terms that were identified in this study are not associated with any reported roles of the DAF-7 pathway, such as Response to Topologically Incorrect Protein. However, TGFβ has been reported to interact with the unfolded protein response in other model systems, such as hepatic stellate cells and corneal endothelial cells, suggesting this function is evolutionarily conserved [41,42]. Regardless of the novelty of the identified GO terms, the DEGs generated from these datasets provide specific gene targets that can be further studied to provide insight into the multifunctional TGFβ pathway.

A limitation of transcriptome studies and the ensuing gene ontology analysis hinges on the robustness of the annotations of the transcriptome database. It is important to keep in mind that as targeted genetic studies elucidate the function of specific genes, the gene ontology analysis will also evolve. In the current study, the presence of expected GO terms, in addition to novel GO categories, suggests that the ontologies are largely on track. However, certain genes, such as the *abu/pqn* genes, while annotated as Response to Incorrect Protein folding, have been reported to function as pharyngeal cuticle proteins [43]. Furthermore, while precautions have been taken to synchronize the samples to minimize the effect of the developmental stage on differential gene expression, it should still be noted that, although the worms were developmentally synchronized, development remains a potential confounding factor in transcriptomic studies. However, qPCR assessment of the selected genes provides validation for the trends seen in our RNA-seq data. Furthermore, we want to emphasize that this study provides a bird’s eye view of the transcriptome. The DEGs identified in this study include those with low FPKM values, though their differential expressions were greater than 2 fold. We retained this subset of genes in order to decrease the likelihood of excluding potentially significant genes, though small subsets of false positive results may also be included. This study focused mainly on the functions of genes that act downstream of both *daf-1* and *daf-3*. The datasets generated in this study will facilitate future investigations into the roles of both the canonical DAF-7 TGFβ pathway as well as genes that are regulated by *daf-1* independent of *daf-3*. While RNA-seq of the *daf-3* single mutants was not included in this study, the differential gene expression provided in the *daf-1* vs. *daf-1; daf-3* and N2 vs. *daf-1;daf-3* (Table S5) tables offers a glimpse of the genes that may be regulated by *daf-3* independent of *daf-1*. Understanding the genes that are downstream of these TGFβ pathway components provides insight into the complexity of this broadly relevant signaling pathway and stimulates the development of new hypotheses regarding its roles in physiologic and pathologic processes.

## Figures and Tables

**Table 1:** GO term enrichment analysis of the genes differentially regulated by *daf-1* and *daf-3.*

Term	Expected	Observed	Enrichment Fold Change	*p* Value	q Value
**Genes downregulated in *daf-1*(*m40*) mutants**					
**Biological Process GO Term**					
Response to biotic stimulus GO:0009607	0.1	3	30	3.20 × 10^−6^	0.00041
**Genes upregulated in *daf-1*(*m40*) mutants**					
**Biological Process GO Terms**					
Response to topologically incorrect protein GO:0035966	1.3	10	7.5	1.30 × 10^−7^	5.50 × 10^−6^
IRE1-mediated unfolded protein response GO:0036498	0.81	4	5	0.0014	0.014
Response to biotic stimulus GO:0009607	1.3	7	5.2	7.10 × 10^−5^	0.0015
Molting cycle GO:0042303	0.77	5	6.5	0.00014	0.0022
**Cellular Component GO Terms**					
Collagen trimer GO:0005581	1.2	30	26	1.10 × 10^−34^	7.10 × 10^−33^
Extracellular region GO:0005576	3.9	16	4.1	5.50 × 10^−7^	1.70 × 10^−5^
Extracellular space GO:0005615	2	7	3.4	0.0011	0.014
Membrane GO:0016020	46	71	1.6	9.20 × 10^−5^	0.0017
Intrinsic component of membrane GO:0031224	40	67	1.7	2.10 × 10^−5^	0.00054
**Molecular Function GO Terms**					
Structural constituent of cuticle GO:0042302	1.1	32	28	4.60 × 10^−38^	5.90 × 10^−36^
Passive transmembrane transporter activity GO:0022803	2.5	8	3.2	0.0011	0.014
Substrate-specific channel activity GO:0022838	2.3	8	3.5	0.00052	0.0074
Peptidase activity GO:0008233	3.1	9	2.9	0.0014	0.014

GO enrichment analysis was performed using the Enrichment Analysis tool on wormbase.org. GO terms were manually separated into the major categories. Arrows denote the child of the parent term immediately above.

**Table 2:** Genes in each GO term associated with biological processes.

Genes Upregulated in *daf-1*(*m40*) Mutants			
**GO Term: Response to Topologically Incorrect Protein Folding GO: 0035966**			
**Gene Name**	**RNAseq Fold Change**	***p* Value**	**q Value**
*abu-1*	6.6291588	0.00015	0.00184372
* abu-8	2.3753599	5.00 × 10^−5^	0.00070927
*abu-10*	2.333622	5.00 × 10^−5^	0.00070927
* abu-11	2.7447639	0.0038	0.0237475
*col-109*	2.7839511	5.00 × 10^−5^	0.00070927
* pqn-74	2.3770804	5.00 × 10^−5^	0.00070927
*fipr-24*	6.4057217	5.00 × 10^−5^	0.00070927
*T06E4.8*	2.1258335	0.0002	0.00233793
*F41E6.11*	2.7376684	5.00 × 10^−5^	0.00070927
*clec-67*	356.70377	0.0007	0.00656909
**GO Term: Response to Biotic Stimulus GO: 0009607**			
**Gene name**	**RNAseq Fold change**	***p* value**	**q value**
*nlp-30*	5.5977832	5.00 × 10^−5^	0.00070927
*fipr-7*	Infinity	5.00 × 10^−5^	0.00070927
*F20G2.5*	4.2163742	5.00 × 10^−5^	0.00070927
*sodh-1	2.0632794	5.00 × 10^−5^	0.00070927
*clec-61	3.9058333	5.00 × 10^−5^	0.00070927
*irg-1*	4.981529	5.00 × 10^−5^	0.00070927
*math-38	8.2638921	5.00 × 10^−5^	0.00070927
**GO Term: Molting Process GO 0042303**			
**Gene name**	**RNAseq Fold change**	***p* value**	**q value**
*dpy-13*	15.645535	5.00 × 10^−5^	0.00070927
*nas-30*	2.0286994	0.0002	0.00233793
*rol-6*	8.4057641	0.0003	0.00330399
*sqt-1*	12.831644	5.00 × 10^−5^	0.00070927
*sqt-2*	5.002505	5.00 × 10^−5^	0.00070927
**Genes downregulated in *daf-1(m40)* mutants**			
**GO Term: Response to Biotic Stimulus GO: 0009607**			
**Gene name**	**RNAseq Fold change**	***p* value**	**q value**
ilys-5	9.66785229	5.00 × 10^−5^	0.00070927
clec-218	5.85451214	5.00 × 10^−5^	0.00070927
lys-4	9.58417075	5.00 × 10^−5^	0.00070927

* represents genes selected for validation by qPCR.

## Supplementary Materials

The following are available online at https://www.mdpi.com/2073-4425/11/3/288/s1. Figure S1: PCA plot of all 9 samples. Table S1: Summary of RNA sequencing analysis. Table S2: List of primers used in qPCR validation. Table S3: List of differentially expressed genes between N2 and *daf-1(m40)*. Table S4: List of differentially expressed genes between *daf-1(m40)* and *daf-1(m40)*; *daf-3(mgDf90)*. Table S5: List of differentially expressed genes between N2 and *daf-1(m40)*; *daf-3(mgDf90)*.
