# Supplementary material for: Transcriptomic Profiling of DAF-7/TGFβ Pathway Mutants in C. elegans
Source: Genes (Basel). 2020 Mar 9;11(3):288. doi: 10.3390/genes11030288 (PMC7140792; doi:10.3390/genes11030288)
Supplement: Supplementary file 1 [file genes-11-00288-s001.zip › supplemental figures 4/Supplemental Figure 1.docx]

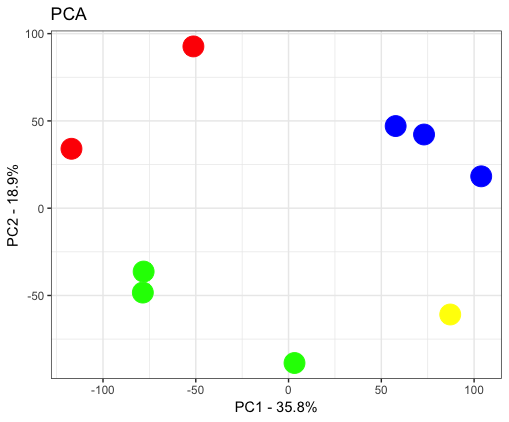


**Supplemental Figure 1:** PCA analysis of all 9 of the sequenced samples. Red dots denote *daf-1;daf-3* (b1, b2) double mutants, blue dots denote *daf-1* single mutants, and green dots denote wild-type N2. Yellow dot denotes *daf-1;daf-3* b3 that was not included in further downstream analysis.
